# New Insights into the *Enterococcus faecium* and *Streptococcus gallolyticus* subsp. *gallolyticus* Host Interaction Mechanisms

**DOI:** 10.1371/journal.pone.0159159

**Published:** 2016-07-27

**Authors:** Ana María Sánchez-Díaz, Beatriz Romero-Hernández, Elisa Conde-Moreno, Young-Keun Kwak, Javier Zamora, Patricia Colque-Navarro, Roland Möllby, Patricia Ruiz-Garbajosa, Rafael Cantón, Laura García-Bermejo, Rosa del Campo

**Affiliations:** 1 Servicio de Microbiología, Hospital Universitario Ramón y Cajal and Instituto Ramón y Cajal de Investigación Sanitaria (IRYCIS), Madrid, Spain; 2 Red Española de Investigación en Patología Infecciosa (REIPI), Sevilla, Spain; 3 Grupo de Biomarcadores y Dianas Terapéuticas, Hospital Universitario Ramón y Cajal and IRYCIS, Madrid, Spain; 4 Microbiology Tumor and Cell Biology Department (MTC), Karolinska Institutet, Stockholm, Sweden; 5 Unidad de Bioestadística Clínica, Hospital Universitario Ramón y Cajal and IRYCIS, Madrid, Spain; University of Florida, UNITED STATES

## Abstract

*Enterococcus faecium* and *Streptococcus gallolyticus* subsp. *gallolyticus* (*S*. *gallolyticus*) were classically clustered into the Lancefield Group D streptococci and despite their taxonomic reclassification still share a similar genetic content and environment. Both species are considered as opportunistic pathogens. *E*. *faecium* is often associated with nosocomial bacteraemia, and *S*. *gallolyticus* is sporadically found in endocarditis of colorectal cancer patients. In both cases, the source of infection is commonly endogenous with a translocation process that launches through the intestinal barrier. To get new insights into the pathological processes preceding infection development of both organisms, we used an *in vitro* model with Caco-2 cells to study and compare the adhesion, invasion and translocation inherent abilities of 6 *E*. *faecium* and 4 *S*. *gallolyticus* well-characterized isolates. Additionally, biofilm formation on polystyrene, collagen I and IV was also explored. Overall results showed that *E*. *faecium* translocated more efficiently than *S*. *gallolyticus*, inducing a destabilization of the intestinal monolayer. Isolates Efm106, Efm121 and Efm113 (*p* < .001 compared to Ef222) exhibited the higher translocation ability and were able to adhere 2–3 times higher than *S*. *gallolyticus* isolates. Both species preferred the collagen IV coated surfaces to form biofilm but the *S*. *gallolyticus* structures were more compact (*p* = .01). These results may support a relationship between biofilm formation and vegetation establishment in *S*. *gallolyticus* endocarditis, whereas the high translocation ability of *E*. *faecium* high-risk clones might partially explain the increasing number of bacteraemia.

## Introduction

*Enterococcus faecium* and *Streptococcus gallolyticus* subsp. *gallolyticus* (*S*. *gallolyticus*) are Gram-positive inhabitants of the human and animal gastrointestinal tracts. At the beginning of the past century, they were clustered together into the Lancefield Group D streptococci based on the presence of a glycerol teichoic acid on their membranes. In 1984, DNA-DNA hybridization studies and 16S rDNA nucleotide sequencing supported the excision of these bacteria into the newly established genus *Enterococcus*, grouping the ancient *Streptococcus faecalis* and *Streptococcus faecium* species, and the *Streptococcus bovis* group [1]. Over the last decade, the *S*. *bovis* group has been reclassified into different species and subspecies, of which the most clinically relevant species in humans is *S*. *gallolyticus* [2]. Despite these taxonomic changes, *E*. *faecium* and *S*. *gallolyticus* species still share metabolic routes, ecological environments and have a similar genetic content [3]. Moreover, they may act both as opportunistic pathogens, mainly in immunocompromised patients, but in different clinical contexts: *E*. *faecium* is often associated with nosocomial invasive infections mainly in oncohaematological, organ transplant, dialysed and intensive care-admitted patients [4,5], whereas *S*. *gallolyticus* bacteraemia and or endocarditis has been strongly associated with the existence of colorectal cancer[6].

In recent years, *E*. *faecium* has emerged as a relevant nosocomial pathogen and is one of the most common causes of bacteraemia in European hospitals [7,8]. This increase in prevalence is related with the worldwide spread of a successful multidrug resistant hospital-adapted lineage, formerly Clonal Complex 17 (CC17) clustered by *Bayesian analysis of population structure* (BAPS) into subgroups BAPS 2.1a and BAPS 3.3a [9]. These high-risk clones are frequently enriched in putative virulence determinants such as enterococcal surface protein (*esp*_Efm_), hyaluronidase-like protein (*hyl*_*Efm*_) or collagen adhesin (*acm*_*Efm*_) among others [10]. On the contrary, *S*. *gallolyticus* is frequently associated with rural areas and livestock [11,12] and its virulence is related to its adherence ability (Pil1 pilus) and biofilm formation [13,14].

*E*. *faecium* and *S*. *gallolyticus* bacteraemia and endocarditis usually represent the final consequence of an endogenous process starting with gut translocation. This process may be favoured by some pathological conditions such as pancreatitis, trauma, surgery or cytotoxic drugs that lead to the increase of the gut barrier permeability [15]. In colorectal cancer and oncohaematological patients, the intestinal epithelium integrity is compromised and thus, permeability alterations and damage to the mucus layer are frequently observed [13,16]. In the present study, we used an *in vitro* cell culture model with Caco-2 cells to examine the differences in translocation ability through an intact epithelium of clinical and commensal strains of *S*. *gallolyticus* and *E*. *faecium* from different origins. Additionally, the adhesion and invasion properties and their ability to form biofilms on different surfaces were also determined.

## Material and Methods

### Bacterial strains

We used a collection of 6 *E*. *faecium* and 4 *S*. *gallolyticus* well-characterized strains causing bacteraemia/endocarditis and gut colonization (CEIC-106/09) (Table 1).

**Table 1:** Main characteristics of the strains and summary of their translocation, adhesion, invasion and biofilm formation ability.

Strain	Source	Source	MLST (ST)	Antibiotic resistance	Other features	% TER/ basal TER (min-max)	Translocation		Adhesion	Invasion	Biofilm production			Reference
							Coefficient	Classification			Polystyrene	Collagen-I	Collagen-IV	
Efm106	OH patient	Faeces	18	Amp, HLR-S, Ery	*esp*, *hyl*, *acm*	90.2–103.6	3.70 (2.57–4.82)	High	High	Low	No	Strong	Weak	Sánchez-Díaz *et al*. 2015
Efm113	OH patient	Faeces	117	Amp, Lvx, HLR-S, HLR-G, Ery	*esp*, *hyl*, *acm*	95.4–112.2	3.17 (2.11–4.24)	High	High	Low	No	No	Weak	Sánchez-Díaz *et al*. 2015
Efm121	OH patient	Blood	117	Amp, Lvx, HLR-S, Ery	*esp*, *acm*	98–114	3.70 (2.65–4.75)	High	Medium	Low	No	No	Weak	Sánchez-Díaz *et al*. 2015
Efm197	OH patient	Faeces	117	Amp, Lvx, HLR-S, HLR-G, Ery, Lnz	*esp*, *hyl*, *acm*	96.9–110.8	0.87 (-0.19–1.93)	Medium	Medium	Low	No	Weak	Strong	Sánchez-Díaz *et al*. 2015
Efm217	Outpatient	Faeces	25	Amp, Lvx, HLR-S, Kan, Tet	*acm*	97.02–111.9	2.16 (1.10–3.21)	High	Low	Low	No	Weak	Weak	Tedim *et al*. 2015
Efm222	Outpatient	Faeces	699	--	--	101.3–116.3	--	Medium	Medium	Low	No	No	Weak	Tedim *et al*. 2015
Sg1	Patient	Blood	34	Min	*pil*1	105.7–128.9	NA	Medium	Low	Low	Weak	Strong	Strong	Romero *et al*. 2015
Sg6	Patient	Blood	35	Clin, Fos	*pil*1	103.1–125.2	NA	Medium	Low	Low	No	Strong	Strong	Romero *et al*. 2015
Sg74	Cow	Faeces	24	Hlr-S, Ery, Min, Clin, Sxt	*pil*1	106.4–127.2	NA	Low	Low	Low	Weak	Strong	Strong	Romero *et al*. 2015
Sg78	Calf	Faeces	28	Clin, Sxt, Van, Q/D	--	113.2–128.3	NA	Medium	Low	Medium	Weak	Weak	Strong	Romero *et al*. 2015
Efc29212	ATCC	Control	30	--	--	--	--	--	--	--	Weak	Weak	Strong	www.atcc.org
Lr925	CECT 925T	Control	--	--	--	--	--	No	--	--	--	--	--	www.cect.org

**Abbreviations:** MLST, multilocus sequence typing; ST, sequence type; TER, transepithelial electrical resistance; OH, oncohaematological; Amp, ampicillin; HLR-S, high level resistance to streptomycin, Ery, erythromycin; Lvx, levofloxacin; HLR-G high level resistance to gentamicin; Lnz, linezolid; Min, minocycline, Clin, clindamycin; Fos, fosfomycin; Stx, sulfamethoxazole; Van, vancomycin; Q/D, quinupristin/dalfopristin; *esp*, enterococcal surface protein; *hyl*, glycosyl hydrolase; *acm*, adhesin of collagen of *E*. *faecium; pil*1, Pil1 pilus.

The *Enterococcus faecalis* ATCC 29212 (www.atc.org) and the *Lactobacillus reuteri* (Spanish Type Culture Collection, CECT 925 T) reference strains were used as controls. All strains were grown on Columbia blood agar (Becton, Dickinson, MI, USA) for 24–48 h at 37°C except for *L*. *reuteri* which was cultured under anaerobic conditions on Man-Rogosa-Sharpe agar (MRS, Oxoid, Basingstoke, Hampshire, UK) supplemented with L-cysteine (0.5 g/L) for 48 h at 37°C.

### Intestinal epithelial cell line

We used the human colorectal adenocarcinoma epithelial cell line Caco-2 (European Collection of Cell Cultures) that spontaneously initiates differentiation under normal culture conditions once cells reach confluence [17]. Caco-2 cells were cultured in Dulbecco’s modified Eagle’s minimal essential medium (DMEM high glucose, Gibco, Thermo Fisher Scientific, Waltham, USA) supplemented with 10% foetal Calf Serum (FCS, Thermo Fisher Scientific) and penicillin G-streptomycin-L-Glut (Gibco, Thermo Fisher Scientific) at 37°C with 5% CO_2._

### Translocation assay

Caco-2 cells, seeded at a density of 5x10^4^ cells/well, were cultured onto Transwell^®^ polycarbonate membrane sterile inserts (6.5 mm diameter, 8 μm pore size, Corning, Life Science, New York, USA) until an intact confluent and differentiated monolayer was formed (10–15 days). The monolayer integrity was monitored by measuring the transepithelial electrical resistance (TER) (Millicell ERS-2 Voltohmmeter, Merck Millipore, Darmstadt, Germany) that reflected the tight junctions’ strength. Assays were only performed when TER values were stable and indicative of monolayer differentiation (500–650 Ω/cm^2^, 12–15 days). In additional experiments inmmunofluorescence confocal microscopy was used as a control of Caco-2 cells confluence and differentiation degree on day 15 when TER values reached 500–650 Ω/cm^2^ (S1 Fig).

Monolayers were then washed with PBS and pre-incubated with serum and antibiotic-free DMEM for 2 h and then bacterial inoculum was apically introduced at a MOI of 20. To determine the translocation ability, aliquots from the basolateral compartment were taken at six time points throughout 8 h and were plated onto Columbia blood agar (Difco; Becton Dickinson) or MRS for *L*. *reuteri* (MRS, Oxoid). Plates were incubated 48 h at 37°C and viable bacteria were counted and the number of colony forming units (CFU/ml) was determined. TER was monitored during the performance of each experiment before every sampling point and data were expressed as percentages of pre-infection TER and post-infection TER measurements. The strain translocation ability was classified as low (1–2 logs), medium (2–4 logs) or high (>4 logs) based on the final number of the translocated bacteria (CFU/ml at t = 8 h).

### Cell adherence and invasion assay

Bacterial adherence and invasion over the intestinal epithelium was assessed *in vitro* as previously described [18]. Briefly, Caco-2 cells (passages 3–15), seeded at a density of 1x10^5^ cells/well and were cultured on 24 well-plates (Corning, Life Science) for 10–14 days. After PBS (Gibco, Life Technologies) washing and serum and antibiotic-free DMEM replacement, cells were infected at a MOI of 20. After 2 h of incubation at 37°C and 5% of CO_2_, monolayers were washed 3 times with pre-warmed PBS to remove non-adherent bacteria and then lysed in 0.1% v/v triton-x100/PBS (Sigma-Aldrich, USA). For the epithelial cell invasion assay, after bacterial incubation and subsequent washing with PBS, extracellular adherent bacteria were killed after 1 h of incubation with 200 μg/ml streptomycin and 50 μg/ml ampicillin for *S*. *gallolyticus* and 10 μg/ml vancomycin and 100 μl/ml lysozyme for *E*. *faecium*.

Thereafter monolayers were washed thrice with PBS and lysed with 1% Triton-X-100/PBS for 15 min. Antibiotic killing efficacy in DMEM was tested previously for all strains employing 10^7^ CFU/ml inoculum (data not shown). The adherent/invasive bacteria were determined after counting CFUs of 10-fold serial dilutions plated on Columbia blood agar. Adherence and invasion were expressed as a percentage of the inoculum.

### Biofilm formation

The ability of each isolate to form biofilm was evaluated as previously described [19] with minor modifications. Briefly, bacterial isolates were cultured overnight at 37°C in Brain Heart Infusion broth (BHI) (Becton, Dickinson) and diluted in BHI to 10^7^ CFU/ml. Polystyrene 96-well microplates, uncoated and coated with collagen type I (rat tail, Corning BioCoat, Thermo Fisher Scientific), or type IV (mouse, Corning BioCoat, Thermo Fisher Scientific), were inoculated with 100 μl of these bacterial suspensions. After 18 h of dynamic incubation (30 r.p.m.) at 37°C, the plates were washed 3 times with PBS and dried 1 h at room temperature. Biofilms were stained with 1% crystal violet (Panreac, Barcelona, Spain) for 15 minutes then washed and air-dried. The biofilm-associated dye was solubilized in 100 μl of ethanol-acetone (75:15 v/v) and absorbance at 600 nm (OD_600_), representative of the amount of biofilm formed, was determined using an automatic spectrophotometer. The wells exposed only to medium without bacteria were used as negative controls. Strains were classified as follows: non-biofilm formers, OD_600_<0.120; weak formers, OD_600_≤ 0.240; and strong formers, OD_600_>0.240 [19]. All the above described experiments were performed in triplicate in at least three independent experiments.

### Statistical analysis

Translocation experiments were analysed using multilevel mixed linear regression models. Random effects were estimated for the variables the day of the experiment (n = 3) and replication (n = 15 for every isolate), which were considered clustering levels. Average translocation, expressed as CFU/ml, was transformed logarithmically to achieve a normal distribution. This log-transformed variable was then fitted using isolate, time and percentage of TER over basal TER as fixed effects of this model. For all analyses we considered 5% as the statistical significance level and results were referred to isolate Efm222. Biofilm formation (means OD_600_) and adhesion/invasion data were compared using the Mann-Whitney U-test. These statistical analyses were performed with STATA^™^ software version 11.0 (StataCorp LP, Texas, USA).

## Results

### *E*. *faecium* strains translocate more efficiently

**Fig 1:**
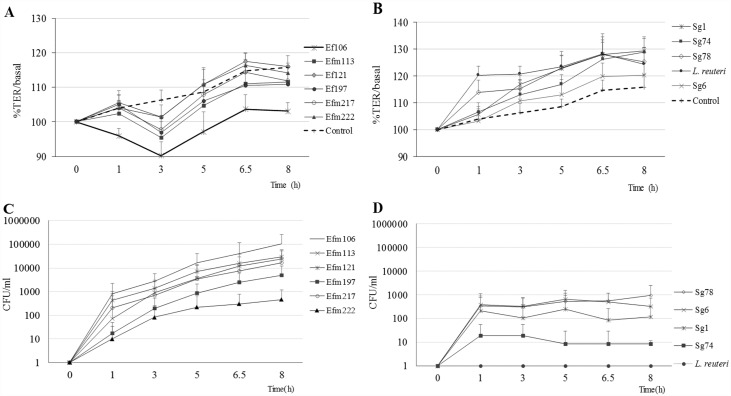
***E*. *faecium* (A) and *S*. *gallolyticus* (B) TER response**. TER values were monitored before and after infection throughout an 8 h period and results were expressed as percentages of mean TER (±SD) at each time point in relation to basal value (before infection). ***E*. *faecium* (C) and *S*. *gallolyticus* (D) translocation across an epithelial monolayer.** Bacterial translocation was expressed as mean (± SD) colony forming units (CFU/ml). Translocation results for *E*. *faecium* strains were compared to Efm222, *p< .05. These figures summarize the results of three independent experiments comprising 3–6 replicates of each isolate.

All *E*. *faecium* strains, except the Efm222 isolate recovered from gut colonization, were able to cross through Caco-2 cells more efficiently than the *S*. *gallolyticus* strains (Fig 1). Indeed, the translocation ability of the four *S*. *gallolyticus* isolates was considered low (Sg74) or medium (Sg1, Sg6 and Sg78) whereas *E*. *faecium* isolates were classified as medium (Efm222) or high (Efm197, Efm217, Efm113, Efm121, Efm106). The *L*. *reuteri* strain was unable to translocate in any of the experiments.

The TER estimations for the Caco-2 monolayer integrity and cell differentiation were confirmed by immunofluorescence of ZO-1, E-cadherin, polymerized actin (phalloidin) and paxillin (S1 Fig). TER monitoring consistently revealed two well-differentiated behaviour patterns after the bacterial inoculum exposition: whereas *S*. *gallolyticus* or *L*. *reuteri* provoked an increase of the TER values, a clear decrease was exhibited by all *E*. *faecium* isolates (Fig 1).

The statistical multilevel regression model only fitted *E*. *faecium* strains behaviour, as several values equal to 0 obtained for *S*. *gallolyticus* strains deviated the model from normality. Differences in the speed of translocation (CFUs/h) of *E*. *faecium* isolates were not found statistically significant, and translocation increased 0.79 logs on average per hour (95% CI: 0.59–0.99) for all *E*. *faecium* isolates (Table 1). For all isolates and at any time, an increase of 1% in the TER value correlated with a decrease in translocation of 0.04 logarithms (*p* = .021) (Table 1).

### *Enterococcus* is more adhesive whereas *S*. *gallolyticus* 78 is highly invasive

**Fig 2:**
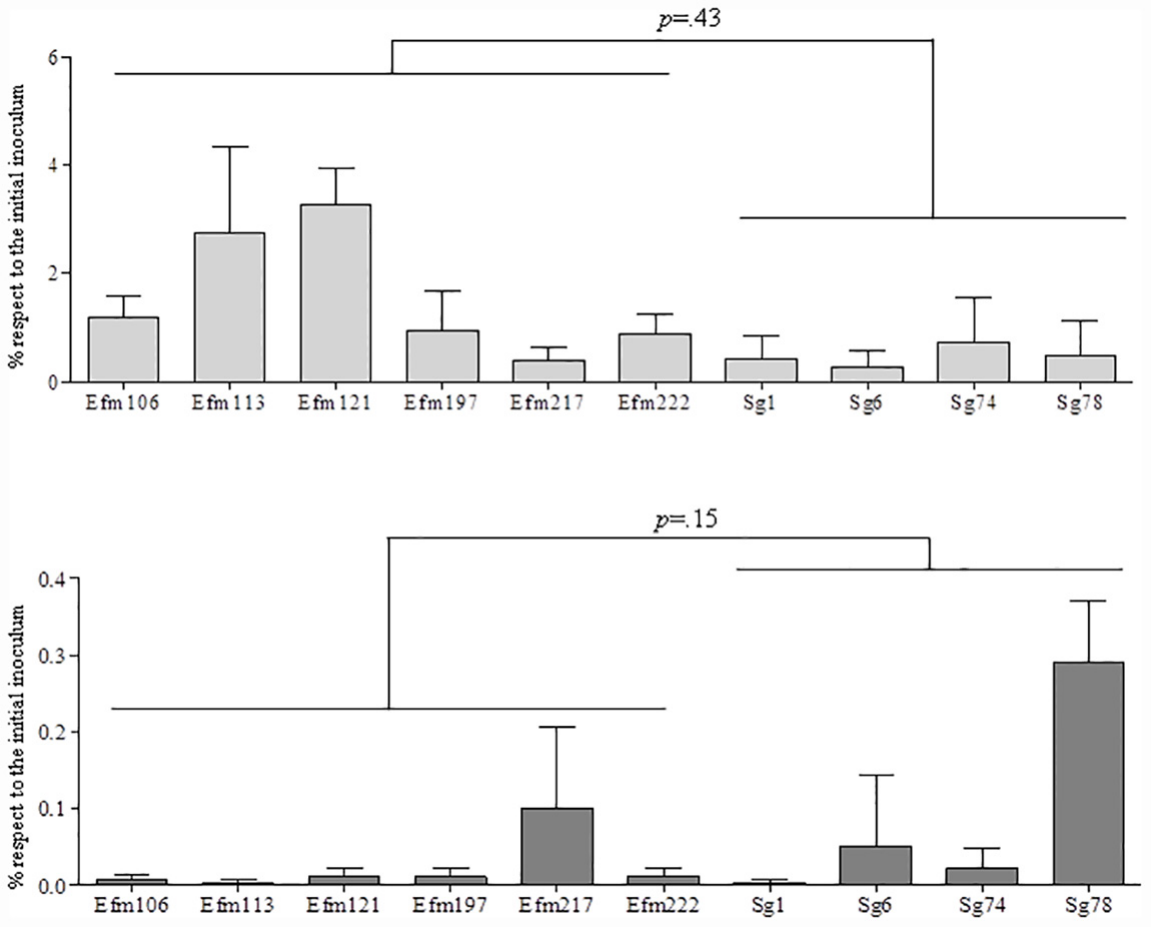
Bacterial adherence and invasion of epithelial Caco-2 cells. Adherence was analysed after 2 h of exposure while the viable internalized bacteria were counted after cell lysis. Results were presented as a percentage of the bacterial inocula and differences between the enterococcal and the streptococcal groups were compared using Mann Whitney U-test.

Overall, *E*. *faecium* strains exhibited better adhesion ability to Caco-2 (cells mean value 1.7%) than the *S*. *gallolyticus* ones (mean value 0.4%), although these differences were not statistically significant (Fig 2). In particular, strains Efm106, Efm113, Efm121 and Efm197, isolated from oncohaematological patients and harbouring the *esp* and *acm* genes, adhered two to six times more than *S*. *gallolyticus* strains. Invasion ability was not a common trait of the selected isolates. Indeed, invasion was in all cases less than 0.1% of the total inoculum, except for the Sg78 isolate. Despite its low adhesiveness, Sg78 displayed the highest invasion ability (0.28%), followed by Efm217 (0.9%) and Sg6 (0.45%). The remaining isolates showed similar invasion values (0.01% mean value, range 0.01–0.02%) (Fig 2).

### Collagen IV is an optimal surface for biofilm formation

**Fig 3:**
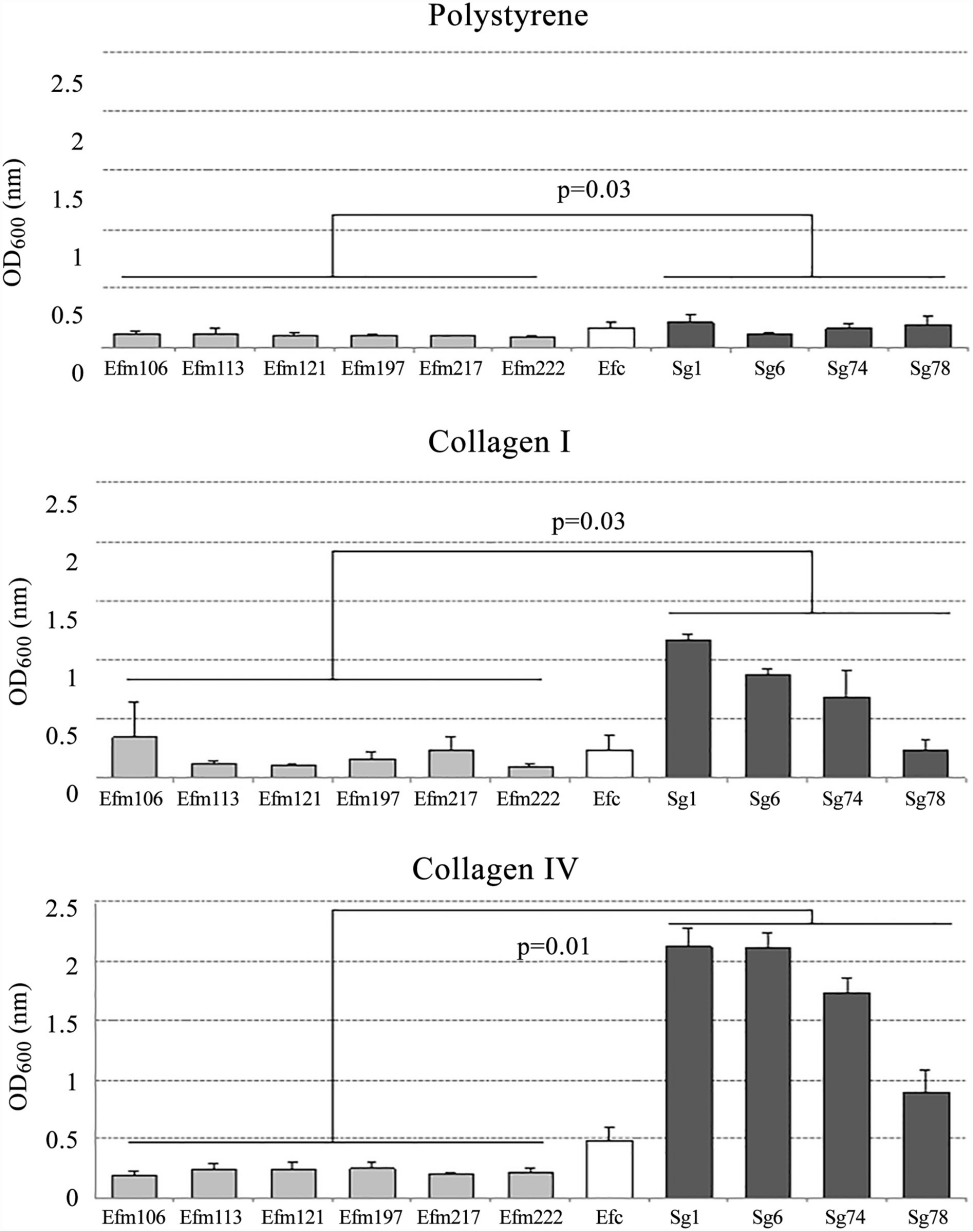
Production of biofilm on polystyrene, collagen I and collagen IV coated surfaces. Isolates were classified as non-biofilm producers (OD_600_<0.120), weak producers (0.120<OD_600_≤0.240) and strong producers (OD_600_>0.240). Results are expressed as mean values (± SD). Biofilm production among the enterococcal and the streptococcal groups was compared using Mann Whitney U-test.

Biofilm production was heterogeneous and differed between species and surfaces (Fig 3). On the three surfaces tested, *S*. *gallolyticus* isolates formed biofilms more efficiently (weak or strong) than the enterococcal ones, independently of their origin. In fact, *S*. *gallolyticus* strains were able to strongly produce biofilm on collagen I (A_600_ 0.23–1.16) and collagen IV (A_600_ 0.89–2.12)-coated surfaces. Globally, the density of the biofilm produced by *S*. *gallolyticus* was, in all cases, significantly higher than that produced by the enterococcal species (Fig 3). On the contrary, the six *E*. *faecium* strains exhibited a similar pattern of biofilm production, absent on polystyrene (0/6); non-formers (3/6) or weak (2/6) on collagen I and weak (5/6) or strong formers (1/6) on collagen IV-coated surfaces, respectively. The control strain *E*. *faecalis* ATCC 29212 was able to form slightly more biofilm on all surfaces than *E*. *faecium*.

The two *S*. *gallolyticus* isolates from bacteraemia (Sg1 and Sg6) formed biofilm on collagen coated surfaces more efficiently than any other strain, although the low number of isolates prevented the association of the source of isolation.

## Discussion

Enterococcal and streptococcal invasive infections are complex and multifactorial processes in which both the host immune system and the bacterial virulence interplay. Most of these infections have an endogenous origin in the intestine lumen, from which bacteria cross the gut barrier by a translocation process. This phenomenon, naturally occurring in healthy individuals at a variable proportion (5–10%), may be further increased under pathological conditions such as ischemic injury, dismotility leading to bacterial overgrowth and states of systemic immunosuppression [15]. Under normal conditions, bacteria crossing the intestinal epithelium are destroyed by phagocytes before reaching the blood circulation, thus preventing the bacteraemic process. Despite the high number of studies dedicated to characterize the gut translocation in Gram-negative bacteria, particularly in *Escherichia coli*, data about Gram-positive bacteria remains scarce. A recent study demonstrated that vancomycin-resistant enterococci are able to translocate at the same level as extended-spectrum beta-lactamase-producing *E*. *coli* (ESBL-*E*.*coli*) [20]. Both, *E*. *faecium* and *S*. *gallolyticus* represent two of the major Gram-positive species with putative gut translocation ability [15,21]; however, the clinical impact differs: *Enterococcus* causes bacteraemia more often, particularly in oncohaematological patients, whereas *Streptococcus* is more commonly involved in infectious endocarditis, almost always in association with colorectal cancer. Based on these observations, we decided to investigate the possible differences between both species concerning their innate ability to translocate since this process could be mainly related to the opportunity to cross a non-preserve barrier, as occurs with *S*. *gallolyticus* during the colorectal cancer [21].

Our results demonstrated that *E*. *faecium* isolates have higher inherent translocation ability than *S*. *gallolyticus*, independently of the origin of the isolates. We also report that the *E*. *faecium* high-risk clones BAPS 2.1a and BAPS 3.3a from oncohaematological patients (blood or faeces) significantly translocated more than the reference colonization Efm 222 isolate, pointing to particularities of each isolate. This high ability to translocate, along with the fact that chemotherapy deeply disrupts the mucous membranes structure [22], could partially explain why *E*. *faecium* bacteraemia in oncohaematological patients is exponentially increasing [7,23] In this group of patients, the administration of antibiotics, such as quinolones or third generation cephalosporins, also leads to a reduction in gut microbiota diversity that contributes to the *E*. *faecium* overgrowth [24]. Several studies highlight the enterococcal dominance [25,26] and others have documented that this overgrowth precedes and may even increase up to 9-fold the risk of vancomycin-resistant *E*. *faecium* blood stream infection [24,27].

In our model, *E*. *faecium* induced a decrease in TER values below that of the control cells (not exposed to bacteria) suggesting a destabilization of the epithelium. The monitoring of the TER values has been proved to be an accurate quantitative technique to measure the integrity of tight junction dynamics in cell culture models of endothelial and epithelial monolayers. Thus, changes in the TER values have been related to the integrity/functionality of the paracellular occluding barrier [28]. Our results confirm that increases in the TER value correlated with a decrease in the *E*. *faecium* translocation rate, pointing to a paracellular route as the main translocation path. However, further determinations to quantify the expression and the localization of the main tight junction’s components, ZO-1 and occluding, during and after bacterial exposure are required.

On the contrary, the Caco-2 monolayer cells reacted to the *Lactobacillus* and *S*. *gallolyticus* insults increasing the TER values. Similar findings have also been reported previously and seem to be related to a probiotic-like effect [29,30].

*S*. *gallolyticus* is a commensal inhabitant of the human and animal gastrointestinal tracts. However, in healthy humans the faecal carriage rate is much lower than that found in ruminants, ranging from 5–10% in adults, and is even higher in neonates [31]. Differences in gut colonization rates might be influenced by variances in adhesion abilities of the colonizing strains and by the epithelium adhesion proteins expression since the effect of bacterial inoculum on the adhesiveness/invasiveness has been ruled out by others [32].

In our work, all *S*. *gallolyticus* isolates, except the vancomycin-resistant Sg78 strain, exhibited low adhesiveness and had non-invasive ability. Results comparable to ours (invasion<0.02%) were previously reported by Boleij and colleagues when human colorectal adenocarcinoma (Caco-2 and T-29) cell lines were used for the experiments [13]. However, when assays were conducted on endothelial cell lines (primary, HUVEC, or highly differentiated, EA.hy926) higher invasion rates (0.1–10% of adherent cells) were found [32]. This fact could suggest that bacterial invasiveness may also vary with cell substrates.

The *S*. *gallolyticus* 78, the most invasive isolate, was included due to its unusual resistance to vancomycin [11]. However, the influence of this trait could not be assessed, as we had not compared it with a vancomycin susceptible isogenic strain.

Recent studies have revealed the importance of the cellular adhesion of *E*. *faecium* and *S*. *gallolyticus* by different microbial surface components recognizing adhesive matrix molecules (MSCRAMMs) [33,34]. These components represent a particular class of proteins expressed on the surface of these species that mediate bacterial binding to host serum and extracellular matrix proteins leading to firm attachment. Variations in the expression of these components might also modulate the translocation process and be responsible for the differences detected in each strain. In *Enterococcus* spp., one of the best characterized is the enterococcal surface protein gene, *esp*, located on a pathogenicity island in both *E*. *faecalis* and *E*. *faecium*. In *E*. *faecium* the presence of *esp* has been related with initial adherence to polystyrene and biofilm formation in urinary experimental infection models [35]. Moreover, *esp* is an important marker in epidemic strains, as is the case of four of our isolates, since its presence seems to be limited to hospital-acquired *E*. *faecium* clones. In our experience, the *esp*^+^ isolates were the most adhesive while displaying a very limited invasive capacity.

Aside from the recently described pathogenicity islands and *pil1* locus [36], detailed studies on the *S*. *gallolyticus* virulence factors remain scarce. This locus, encoding 2 LPXTG proteins (Gallo2178 and Gallo2179) and 1 sortase C (Gallo2177), was shown to be essential for adhesion to collagen I and contributed to colonization and establishment of infective endocarditis in rat models [14]. In our model, the fact that three out of four *S*. *gallolyticus* strains presented the *pil*1 gene was not associated with an enhanced adhesiveness, and it was Sg78, the isolate lacking this operon, the most adherent and invasive isolate.

This lack of correspondence might be explained by the fact that the presence of virulent determinants is not always linked with their expression *in vivo*, and the possibility of down-regulation or merely the lack of expression should be taken into account for future studies.

The main limitation of our study is that we tested a low number of strains which impeded us to compare translocation, adhesion and invasion abilities of the strains considering the source and the intra-species particularities. However, the complex methodology of the experiments determined the number of strains, and we focused on the origin of the strains: commensal and bacteramic/endocarditis isolates.

Biofilm formation is a well- recognized virulence and antibiotic resistance factor and its expression has been related to colonization of a variety of medical devices (catheters, prosthetic heart valves or orthopaedic appliances) and is associated with several human diseases, such as native valve endocarditis or burn wound infections among others [37]. The high ability of *S*. *gallolyticus* to produce biofilm on collagen-rich surfaces was previously described, particularly on collagen I and IV [13]. These molecules are not usually accessible but in damaged heart valves or in polyps and early colorectal tumors they can be exposed. The high ability of *S*. *gallolyticus* strains to form biofilm on these surfaces may partially explain their relation with this clinical illness.

In previous studies, *E*. *faecium* was found to produce biofilm in a lesser extent than *E*. *faecalis* although its production was more frequently associated with clinical isolates carrying *esp* gene, rather than environmental or from healthy individuals [19,38]. In our work, we report similar observations but correlation with *esp* presence was not found. Differences in the biofilm production could be also related to the absence of a supplemented carbohydrate in the growing medium [19,39].

In our tertiary hospital (≈800 beds), during the period 2005–2014 we found that *E*. *faecium* caused two fold more cases of bacteraemia than of endocarditis (0.02 *vs*0.01%), whereas the opposite situation was observed for *S*. *gallolyticus*, which caused eight times more endocarditis than bacteraemia (0.04 *vs* 0.005%) (unpublished data). The main results of our work might explain these inter-species clinical differences: *S*. *gallolyticus* forms more biofilm favouring the endocarditis vegetation establishment, whereas *E*. *faecium* is more invasive which supports the bacteraemia occurrence. In summary and despite the high genetic and biochemical similarities of both species, the results obtained in this work highlight their gut translocation abilities differences.

## Supporting Information

S1 Fig Assessment of Caco-2 monolayer integrity by immunofluorescence staining of ZO-1, E-cadherin, Paxillin and Phalloidin and confocal microscopy. A) Immunofluorescence of E-cadherin (red) and ZO-1 (green) observed by confocal microscopy. The orthogonal projection indicates that E-cadherin is uniformly distributed in the cell membrane and that ZO-1 is punctually located above the E-cadherin staining. On the right, the contrast phase image shows the monolayer confluence. B) Immunofluorescence of Paxillin (green) and polymerized actin staining (phalloidin) at cell middle (upper image) and basal level (lower image). The upper orthogonal projection image shows the actin ring and the microvilli whereas the actin stress fibres are displayed in the lower image. Paxillin staining co-localized with actin at the cell basal level, indicating the assembly of FAC (Focal Adhesion Complexes).
(DOCX)
