# Supplementary material for: New Insights into the Enterococcus faecium and Streptococcus gallolyticus subsp. gallolyticus Host Interaction Mechanisms
Source: PLoS One. 2016 Jul 27;11(7):e0159159. doi: 10.1371/journal.pone.0159159 (PMC4963119; doi:10.1371/journal.pone.0159159)

**S1-Supporting Information**

**Immunofluorescence confocal microscopy**

Caco-2 cells seeded at 2 x105 cells/well onto coverslips were fixed on day 15 in 4% paraformaldehyde followed by permeabilization with PBS containing 0.1% Triton X-100 for 20 min at room temperature. The cells were blocked with 1% BSA in PBS and reacted with a primary antibody targeting human extracellular E-caherin (mouse, BD), ZO-1 (rabbit, BD) and Paxillin (mouse, BD) 1:100 for 1h at room temperature. After washing steps with PBS, the cells were incubated with appropriate secondary antibodies conjugated with Alexa 488 (anti-rabbit and anti-mouse, Molecular Probes) and Alexa 594 (anti-mouse, Molecular Probes) and phalloidin -Alexa 568 (Invitrogen,1:250) for 1 hour at room temperature. The coverslips were treated with montage medium, mounted onto glass slides and were observed with a confocal microscope (Leica Sp5). Representative images as well as orthogonal projections were acquired with the appropriate Leica Confocal software.

**S1 Fig. Assessment of Caco-2 monolayer integrity by immunofluorescence staining of ZO-1, E-cadherin, Paxillin and Phalloidin and confocal microscopy. A) Immunofluorescence of E-cadherin (red) and ZO-1 (green) observed by confocal microscopy.** The orthogonal projection indicates that E-cadherin is uniformly distributed in the cell membrane and that ZO-1 is punctually located above the E-cadherin staining. On the right, the contrast phase image shows the monolayer confluence. **B) Immunofluorescence of Paxillin (green) and polymerized actin staining (phalloidin) at cell middle (upper image) and basal level (lower image).** The upper orthogonal projection image shows the actin ring and the microvilli whereas the actin stress fibres are displayed in the lower image. Paxillin staining co-localized with actin at the cell basal level, indicating the assembly of FAC (Focal Adhesion Complexes).


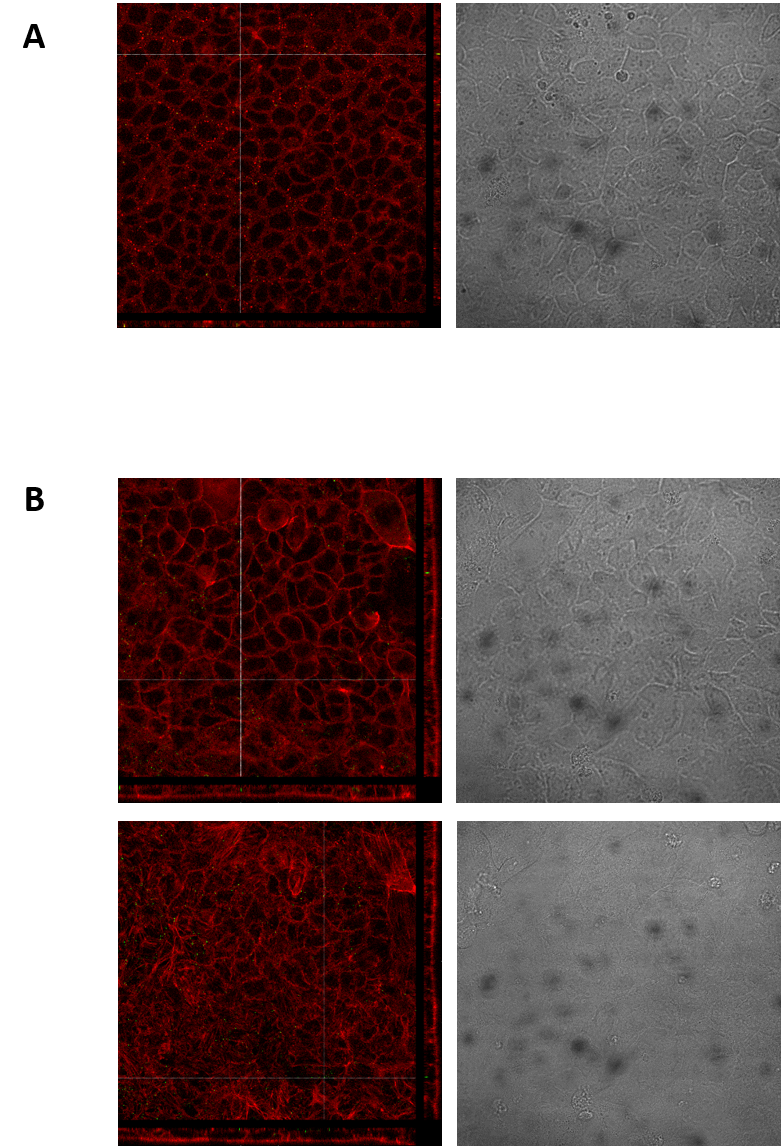

Supplement: S1 Fig — A) Immunofluorescence of E-cadherin (red) and ZO-1 (green) observed by confocal microscopy. The orthogonal projection indicates that E-cadherin is uniformly distributed in the cell membrane and that ZO-1 is punctually located above the E-cadherin staining. On the right, the contrast phase image shows the monolayer confluence. B) Immunofluorescence of Paxillin (green) and polymerized actin staining (phalloidin) at cell middle (upper image) and basal level (lower image). The upper orthogonal projection image shows the actin ring and the microvilli whereas the actin stress fibres are displayed in the lower image. Paxillin staining co-localized with actin at the cell basal level, indicating the assembly of FAC (Focal Adhesion Complexes). (DOCX) [file pone.0159159.s001.docx]
